# 333. Clinical Impact of a Multiplex Rapid Diagnostic Panel in Critically Ill Patients

**DOI:** 10.1093/ofid/ofac492.411

**Published:** 2022-12-15

**Authors:** Jayda Esplund, Alex D Taylor, Tyler Stone, Elizabeth Palavecino, Abdullah Kilic, Vera Luther, James Beardsley

**Affiliations:** Atrium Health Wake Forest Baptist, Winston-Salem, North Carolina; Atrium Health Wake Forest Baptist, Winston-Salem, North Carolina; Atrium Health Wake Forest Baptist, Winston-Salem, North Carolina; Wake Forest School of Medicine, Winston Salem, North Carolina; Wake Forest University, winston salem, North Carolina; Wake Forest School of Medicine, Winston Salem, North Carolina; Atrium Health Wake Forest Baptist, Winston-Salem, North Carolina

## Abstract

**Background:**

The BioFire FilmArray® Pneumonia Panel® (PP) is a rapid diagnostic test that detects select bacterial and viral respiratory pathogens and the presence of certain antimicrobial resistance genes within 75 min of testing. Starting Nov 2021 respiratory specimens from all adult ICU patients (pts) with quantitative cultures (cx) obtained by bronchoalveolar lavage (BAL) or tracheal aspirate (TA) were automatically tested with PP. The purpose of this study was to evaluate the clinical impact of the PP.

**Methods:**

This single-center, retrospective cohort study compared patients prior to (PRE) (Jan - Mar 2021) and after (POST) (Jan - Mar 2022) implementation of the PP. Adult ICU pts with a quantitative cx obtained by BAL or TA during the study periods were reviewed in random order until 25 pts from each study month meeting study criteria were identified (75 pts in each cohort). Pts with another infection other than bacteremia with the same causative pathogen requiring antibiotics (ABX) in the previous 14 days through 5 days after specimen collection or pts who died within 5 days after specimen collection were excluded. ABX therapy for the 5 days after cx collection was evaluated. The primary outcome was time to first ABX change based on microbiologic test results. Secondary outcomes included ABX days of therapy (DOTs), time to adequate therapy, and potential vs actual ABX changes made based on the PP in POST cohort.

**Results:**

PRE pts were a median of 54 yrs old and 56% female compared to 59 yrs and 32% female in POST cohort. Median time to first ABX change based on microbiologic data was 50.3 hrs PRE vs 21.2 hrs POST (p=0.0006). 56 (75%) of POST ABX regimens were eligible for change based on PP results; actual change occurred in 30 (54%) of these regimens (ABX de-escalation in 23 and escalation in 7). Median ABX DOTs were 8 PRE vs 6 POST (p = 0.07). For the pts with ABX changes made based on PP results, median time to first ABX change was 10 hrs. For pts who were initially on inadequate therapy, time to adequate therapy was 67 hrs PRE vs 37 hrs POST.

**Conclusion:**

The PP was associated with decreased time to first ABX change and fewer ABX DOTs. The PP may have had a larger impact if a higher percentage of potential ABX changes were implemented. The PP may be a useful ABX stewardship tool.

**Disclosures:**

**All Authors**: No reported disclosures.

